# Supplementary material for: Traffic jam by GPS: A systematic analysis of the negative social externalities of large-scale navigation technologies
Source: PLoS One. 2024 Aug 6;19(8):e0308260. doi: 10.1371/journal.pone.0308260 (PMC11302916; doi:10.1371/journal.pone.0308260)
Supplement: Attachment — Submitted filename: Response to Reviewers.pdf [file pone.0308260.s007.pdf]

Response to Reviewers for submission PONE-D-23-40588

Dear Reviewers,

We would like to express our sincere gratitude for the valuable feedback provided on our submission (PONE-D-23-40588). We truly appreciate your dedicated time and effort in reviewing our work. In response to your insightful comments, we have carefully revised our manuscript, incorporated the suggestions, and addressed the concerns raised. Firstly, we are pleased to note that the reviewers found our paper well-written [R1] and deemed our dataset intriguing [R2]. These affirmations serve as encouraging validation of our efforts. To facilitate a comprehensive understanding of the revisions made, we have highlighted all major changes in blue within the revised manuscript. Additionally, we have structured a detailed response addressing each of the reviewers' comments, directly referencing relevant sections, figures, and tables in the manuscript.

Literature Review and Context: Regarding the literature review and contextualization, we have augmented the introduction to highlight the consequences of negative societal externalities, thereby strengthening the motivation behind our study. These augmentations are further echoed in the discussion section, providing a coherent narrative throughout the manuscript [R2].

Clarification on the number of articles: In response to the request for clarification on the number of articles reviewed per search term, we have provided explicit details in the designated sections of the manuscript, as suggested by R1.

Updated Tables and Results: Moreover, we have meticulously updated the tables with associated categories and references for individual articles, as per the recommendations of R1. To ensure accuracy and reliability, we have thoroughly reviewed and verified all articles, resulting in a more robust presentation of our findings.

Relationship between negative social externalities and solutions: In addressing the relationship between negative social externalities and the proposed solutions, we have conducted an in-depth analysis, which is visually represented in Figure 3 and described in the paper. This graphical representation offers valuable insights into the effectiveness of various solutions, aligning with the suggestions by R1 and enhancing the coherence of our paper.

Throughout the revision process, we have implemented numerous minor edits to enhance clarity, as suggested by both reviewers. These refinements contribute to the overall readability and coherence of the manuscript. Finally, we have updated all supporting information to align with the revisions made in this iteration. Once again, we thank our reviewers for their thoughtful evaluation and constructive criticism. We trust that the changes will further strengthen our work's quality and impact, and we are looking forward to your response. We believe that our paper contributes to understanding the societal implications of technology by identifying issues like congestion, accidents, and pollution caused by navigation technologies. We emphasize the necessity for collaboration among governments, residents, planners, and technologists to mitigate these negative effects. Additionally, we highlight community and governmental responses, such as street closures and speed limit reductions, to address these challenges.
